# Cell Cycle Re-Entry and Mitochondrial Defects in Myc-Mediated Hypertrophic Cardiomyopathy and Heart Failure

**DOI:** 10.1371/journal.pone.0007172

**Published:** 2009-09-25

**Authors:** Hyoung-gon Lee, Qun Chen, Julie A. Wolfram, Sandy L. Richardson, Anna Liner, Sandra L. Siedlak, Xiongwei Zhu, Nicholas P. Ziats, Hisashi Fujioka, Dean W. Felsher, Rudy J. Castellani, Maria L. Valencik, John A. McDonald, Brian D. Hoit, Edward J. Lesnefsky, Mark A. Smith

**Affiliations:** 1 Department of Pathology, Case Western Reserve University, Cleveland, Ohio, United States of America; 2 Department of Medicine, Case Western Reserve University, Cleveland, Ohio, United States of America; 3 Department of Pharmacology, Case Western Reserve University, Cleveland, Ohio, United States of America; 4 Louis Stokes Cleveland DVAMC, Cleveland, Ohio, United States of America; 5 University Hospitals Case Medical Center, Cleveland, Ohio, United States of America; 6 Division of Oncology, Department of Medicine and Pathology, Stanford University School of Medicine, Stanford, California, United States of America; 7 Department of Pathology, University of Maryland, Baltimore, Maryland, United States of America; 8 Department of Biochemistry, University of Nevada Reno, Reno, Nevada, United States of America; Instituto de Química, Universidade de São Paulo, Brazil

## Abstract

While considerable evidence supports the causal relationship between increases in c-Myc (Myc) and cardiomyopathy as a part of a “fetal re-expression” pattern, the functional role of Myc in mechanisms of cardiomyopathy remains unclear. To address this, we developed a bitransgenic mouse that inducibly expresses Myc under the control of the cardiomyocyte-specific MHC promoter. In adult mice the induction of Myc expression in cardiomyocytes in the heart led to the development of severe hypertrophic cardiomyopathy followed by ventricular dysfunction and ultimately death from congestive heart failure. Mechanistically, following Myc activation, cell cycle markers and other indices of DNA replication were significantly increased suggesting that cell cycle-related events might be a primary mechanism of cardiac dysfunction. Furthermore, pathological alterations at the cellular level included alterations in mitochondrial function with dysregulation of mitochondrial biogenesis and defects in electron transport chain complexes I and III. These data are consistent with the known role of Myc in several different pathways including cell cycle activation, mitochondrial proliferation, and apoptosis, and indicate that Myc activation in cardiomyocytes is an important regulator of downstream pathological sequelae. Moreover, our findings indicate that the induction of Myc in cardiomyocytes is sufficient to cause cardiomyopathy and heart failure, and that sustained induction of Myc, leading to cell cycle re-entry in adult cardiomyocytes, represents a maladaptive response for the mature heart.

## Introduction

c-Myc (Myc) is highly expressed in fetal, proliferating cardiac myocytes. However, soon after birth, myocytes cease to divide corresponding with the downregulation of Myc [1]. Interestingly, while Myc is expressed at very low levels in the adult myocardium under normal physiological conditions, it is upregulated rapidly in response to virtually all hypertrophic stimuli and leads to hypertrophy rather than hyperplasia [2], [3]. Myc encodes a transcription factor that, as part of a heterodimeric complex with MAX, regulates the expression of a multitude of genes involved in regulating cellular proliferation and growth [4]–[7]. Consequently, overexpression of Myc is commonly associated with the activation of cell cycle machinery and tumorigenesis. Myc exerts its neoplastic function by inducing autonomous cellular proliferation and cellular growth, blocking differentiation, and inducing genomic instability [5]–[10].

In various animal models of cardiomyopathy, the upregulation of Myc is a consistent and early change. For instance, Myc mRNA is dramatically increased several hours after the imposition of pressure overload in animal models [1]–[3]. Notably, similar changes in Myc expression have been reported under other pathological circumstances including myocardial infarction and hypertrophy induced by pharmacological stimulation [11], [12]. In humans, Myc mRNA is increased in hypertrophic cardiomyopathy patients [13]. While the ability to detect chronological changes in humans is limited because samples are obtained postmortem or at biopsy, the consistent detection of Myc expression raises the question of whether the early expression of Myc is necessary or sufficient to induce disease. Moreover, if Myc expression is sufficient to cause disease, it is unclear which Myc-related downstream mechanisms, such as cell cycle re-entry, contribute to cardiomyopathy. In this regard, given that mitochondrial alterations are directly linked to the development of cardiomyopathy and heart failure [14]–[17], it is interesting to note that Myc also plays a role in the maintenance of mitochondrial function [18]. While the mechanism of Myc-regulated mitochondrial function is unclear, the regulation of PGC-1α (PPARγ coactivator-1α) appears to be a key factor in this process since Myc induced the biogenesis of mitochondria through a PGC-1α-related pathway [18]. PGC-1α is a major regulatory molecule of mitochondrial proliferation and induces synthesis of both mitochondrial DNA-encoded and nuclear-encoded mitochondrial peptides. Importantly, the expression of PGC-1α is directly associated with the development of cardiomyopathy [19], [20]. Thus, Myc may induce hypertrophic cardiomyopathy though reactivation of the cell cycle machinery and by negatively affecting mitochondrial function. To test this hypothesis, using a newly developed inducible transgenic mouse model, we examined the effect of Myc on the development of cardiomyopathy and heart failure and mechanistically evaluated alterations in cell cycle and mitochondria.

## Results

### Establishment of MHC-Myc mice

**Figure 1:**
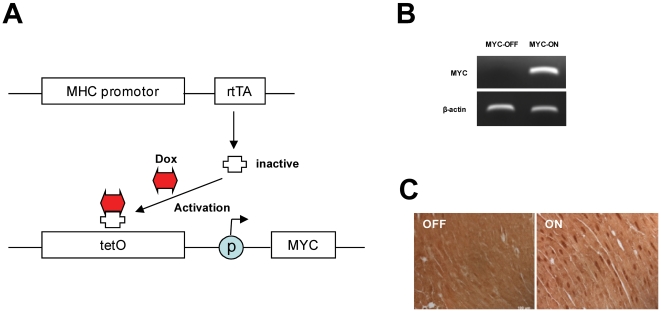
The regulation of Myc in MHC-Myc mice. (A) The rtTA molecule is driven by MHC promoter. rtTA requires doxycycline (Dox) for binding to tetO sequence to drive expression of Myc. Without Dox, rtTA remains inactive and Myc expression is not initiated. (B) mRNA level of Myc is measured by RT-PCR after 1 week doxycycline diet. In comparison to the basal level (Myc-OFF). (C) The level of protein expression is analyzed by human Myc specific rabbit monoclonal antibody. The expression of Myc is strongly induced in nuclei of cardiomyocytes by doxycycline diet after 1 week (ON) but no expression of Myc is detected in control mice (OFF).

Traditional transgenic mouse models have significant limitations for studying the pathogenic mechanism(s) of cardiomyopathy due to adverse effects of the transgene in development and also limitations in studying the temporal effect of a given transgene in mice that constitutively overexpress the transgene. To circumvent both of these problems and to study the importance of Myc-driven cell cycle re-entry in the pathogenesis of cardiomyopathy, we used a tetracycline-controlled system to generate bitransgenic mice (MHC-Myc) that inducibly overexpress Myc under the control of the αMHC gene promoter that drives high transgene expression specifically in cardiomyocytes [21]. To generate MHC-Myc mice, we mated the transgenic line tet-o-Myc, which contains the tetracycline response element adjacent to Myc cDNA [9], with the transgenic line αMHC-rtTA (JAM8585), which drives the expression of rtTA in cardiomyocytes. rtTA requires doxycycline to bind tetO and, therefore, the transcription of Myc is activated by doxycycline (Figure 1A). The MHC-Myc mice are derived from heterozygote crosses and, as a result, 75% of the litter is, as expected, composed of non-bitransgenic mice that serve as controls (+/− doxycycline). We have not experienced any issues in the breeding of, nor maintenance, of these mice which remain healthy unless the transgene is activated.

To examine the inducible expression of Myc, doxycycline (200 mg/kg) containing diet was provided for 1 week and hearts were collected for the measurement of Myc expression. Mice possessing both transgenes and on doxycycline diet exhibited increased expression of Myc mRNA (Figure 1B) and protein (Figure 1C) compared with the basal level of Myc expression in similar animals that were not fed doxycycline. After 1 week of induction by doxycycline, prominent expression of Myc in the nuclei of cardiomyocytes was detected using an anti-Myc specific rabbit monoclonal antibody. These data clearly demonstrate that Myc expression can be tightly regulated by the doxycycline diet and that there is little/no significant leakiness of Myc expression in the MHC-Myc mice.

### Hypertrophy and heart failure by Myc induction

**Figure 2:**
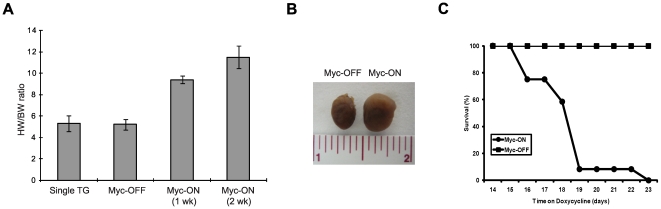
Increase of Myc expression in adult cardiomyocytes induces cardiomyopathy and heart failure. (A) Measured heart weights (mg) were normalized to body weight (g). 1 week Myc-ON mice demonstrate about 80% increase of normalized cardiac mass and great than 100% increase is observed after 2 weeks and the dramatic increase of cardiac mass is observed in isolated hearts (B). (C) Myc-ON mice exhibit increased mortality after 2 weeks of Myc induction while no lethality was observed in Myc-OFF mice (n = 12).

We used 3–4 month old MHC-Myc mice to determine the effect of Myc on adult cardiomyocytes. At baseline, hearts from MHC-Myc mice were indistinguishable in terms of size, histology or echocardiography from littermate control mice. However, the overexpression of Myc, with doxycline-diet in Myc-ON mice, resulted in 100% mortality in 3 weeks (Figure 2C) wherease no mortality was observed in Myc-OFF nor littermate control mice up to 14 months old, which are the oldest MHC-Myc mice in our current colony.

To characterize the phenotype in the adult Myc-ON mice, cardiac structure and function were assessed by echocardiography after 2 weeks of doxycycline administration (female, 3–4 month old, n = 8 Myc-OFF, n = 5 Myc-ON). Cardiac-restricted temporally-defined overexpression of Myc resulted in a marked increase in LV mass with concentric geometry (increased relative wall thickness), reduced LV fractional shortening, decreased cardiac output, increased myocardial performance index (indicating impaired contraction and relaxation), and left atrial enlargement, suggesting an increase in left atrial pressure (Table 1). Thus, Myc overexpression for 2 weeks produced decompensated hypertrophic cardiomyopathy characterized by impaired systolic and diastolic function. Importantly, single transgenic control mice, which only have either the Myc or the MHC-rtTA gene, on a similar doxycycline diet showed no significant changes in any of the parameters measured.

**Table 1:** Echocardiographic Analysis of MHC-Myc mice.

	Myc-OFF	Myc-ON
AoR (cm)	0.15±0.01	0.14±0.02
Left atrial (cm)	0.23±0.02	0.34±0.05*
myocardial performance index	0.36±0.07	0.91±0.34
IVSd (cm)	0.10±0.01	0.14±0.02*
PWTd (cm)	0.10±0.01	0.12±0.02*
Cardiac output (cc)	0.28±0.07	0.13±0.08*
EDD (cm)	0.38±0.02	0.40±0.05
ESD (cm)	0.23±0.03	0.28±0.03*
Fractional shortening	0.53±0.10	0.28±0.06**
LVMI (mg/g)	6±1	13±4**
Relative wall thickness	0.53±0.16	0.65±0.06**

* p<0.05.

** p<0.001.

All data are expressed as the mean±SD. Abbreviations: aortic root diameter (AoR), end diastolic dimension (EDD), end systolic dimension (ESD), interventricular septal thickness in diastole (IVSd), left ventricular mass index (LVMI), posterior wall thickness in diastole (PWTd).

**Figure 3:**
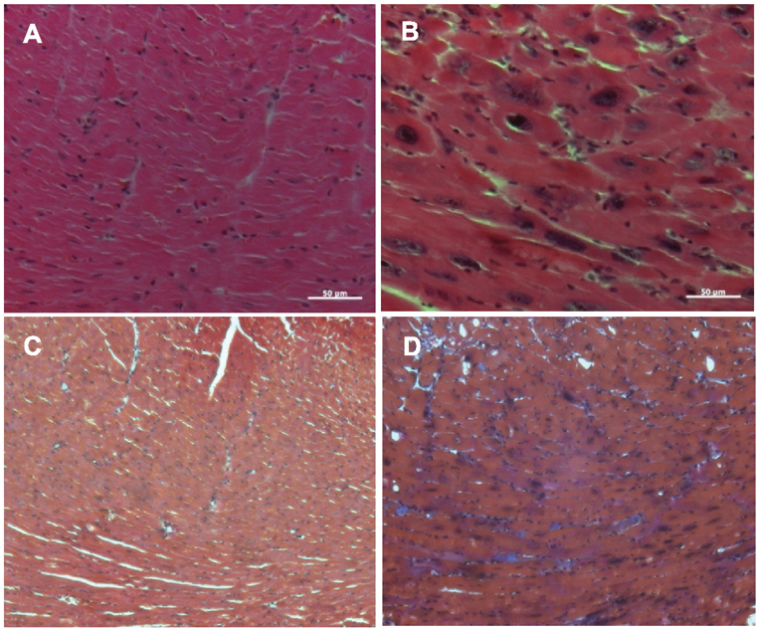
Histological analysis of hearts. Myocardial sections from Myc-OFF (A, C) and Myc-ON (B, D) were stained with hematoxylin and eosin (A, B). Significant myocardial disarray is observed in Myc-ON mice (B). Trichrome staining (C, D) also reveals hypertrophied cardiac myocytes in Myc-ON mice with mild fibrosis (D). Original magnification: X 400 for A and B, X 200 for C and D. Scale bar = 50 µm.

**Figure 4:**
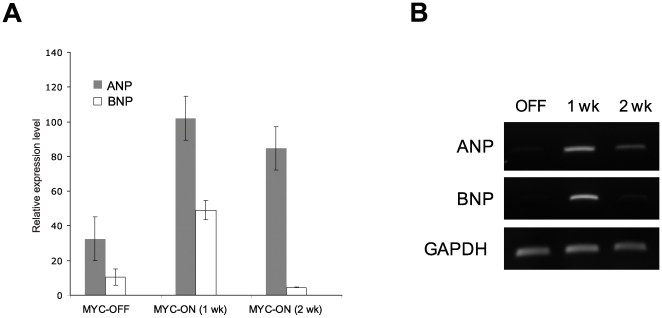
The change of molecular markers for cardiac hypertrophy by over expression of Myc. Total ventricular RNA from mice with indicated Myc induction duration was analyzed by RT-PCR with specific primers. Each gene expression level was quantified and normalized with GAPDH expression level and expressed as a relative intensity.

Consistent with the echocardiographic data, a two week induction of Myc resulted in an approximately 2-fold increase in gravimetric cardiac mass normalized to body weight compared with Myc-OFF mice (Figure 2A,B). Histological analysis revealed typical hypertrophic changes including myocardial disarray, nuclear atypia and marked increase in fiber width (Figure 3A–C) as well as interstitial deposition of collagen (Figure 3D). One of the most conserved features of cardiac hypertrophy is the re-activation of genes that are normally limited in their expression to the embryonic stage of heart development and these are often used as molecular markers for cardiac hypertrophy. Therefore, we examined hypertrophic changes at the molecular level by measuring two well known marker genes, atrial natriuretic peptide and brain natriuretic peptide. As shown in Figure 4, the expression of both atrial and brain natriuretic peptides was significantly increased after 1 week induction and then decreased after 2 weeks induction. This suggests that hypertrophic changes at the molecular level begin early in the time course of Myc expression and that these changes are temporarily regulated.

### Cell cycle re-entry in Myc-ON mice

**Figure 5:**
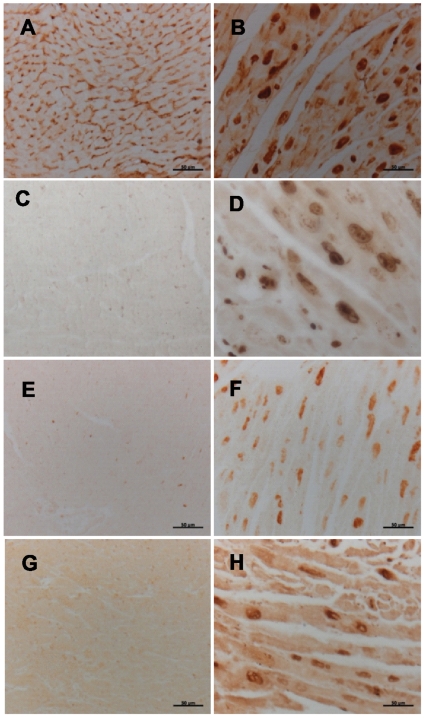
Expression of cell cycle markers in the cardiomyocytes from Myc-ON mice. The expression of PCNA, Ki67 and cyclin D1 is dramatically increased in the nuclei of cardiomyocytes from Myc-ON mice (B, D, F). In Myc-OFF mice, no PCNA, Ki67 and a basal level of cyclin D1 is observed (A, C, E). DNA replication in cardiomyocyte in Myc-ON mice is confirmed by BrdU incorporation (H) compared with Myc-OFF mice (G). Original magnification: X 400. Scale bar = 50 µm.

To test whether Myc expression induces cell cycle re-entry in adult cardiomyocytes, we measured the expression level of PCNA, Ki67, and cyclin D1, which increase during the cell cycle. We found that the expression of PCNA, Ki67, and cyclin D1 was strongly and specifically induced in cardiomyocytes in Myc-ON animals (Figure 5). In quantification analysis, we found that 38±2.8% of cardiomyocyte nuclei in Myc-ON animals were positive for Ki67. To confirm such aberrant cell cycle activity, we injected BrdU (50 mg/kg bodyweight) intraperitoneally 24 hrs before sacrificing the mice and DNA replication was measured by BrdU incorporation. Consistent with our PCNA, Ki67, and cyclin D1 data, a significant number of cardiomyocyte nuclei (23±2.8%) from Myc-ON mice were labeled with anti-BrdU antibody although no significant BrdU incorporation was observed in Myc-OFF mice (Figure 5E,F). Taken together, these data suggest that the cell cycle machinery is activated in cardiomyocytes after Myc expression.

### Apoptosis is increased in Myc-ON mice

**Figure 6:**
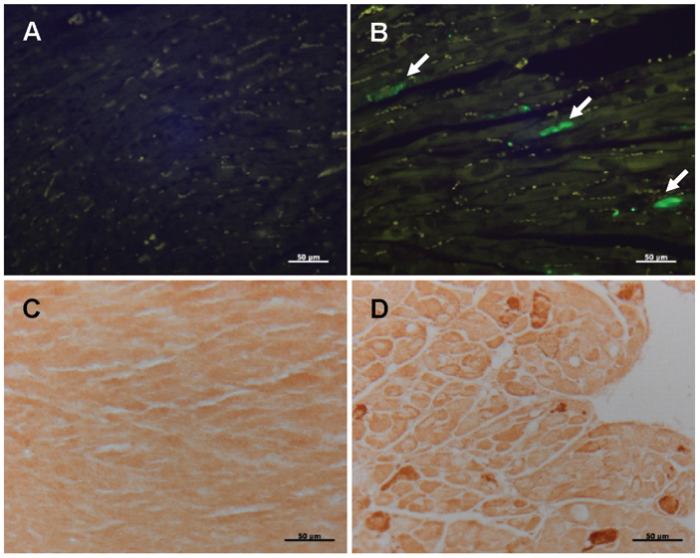
Increased apoptosis in Myc-ON mice. TUNEL positive nuclei (arrows) are specifically localized in the cardiomyocyte nuclei of Myc-ON mice (B) but not in Myc-OFF mice (A). The active form of caspase-3 was detected by anti-cleaved caspase-3 antibody and the increase number of positive cells is found in Myc-ON mice (D) but not in Myc-OFF mice (C). Original magnification: X 400. Scale bar = 50 µm.

We next examined the pathological effects of induction of Myc on apoptosis in cardiomyocytes. By comparing TUNEL staining in Myc-ON and Myc-OFF mice, TUNEL positive cardiomyocytes were increased in Myc-ON mice but not in Myc-OFF mice (Figure 6A,B). Consistent with this, the active form of caspase-3, which is increased in apoptotic cells, was also increased in Myc-ON mice (Figure 6C,D).

### Ultrastructural and functional alteration in mitochondria in Myc-ON mice

**Figure 7:**
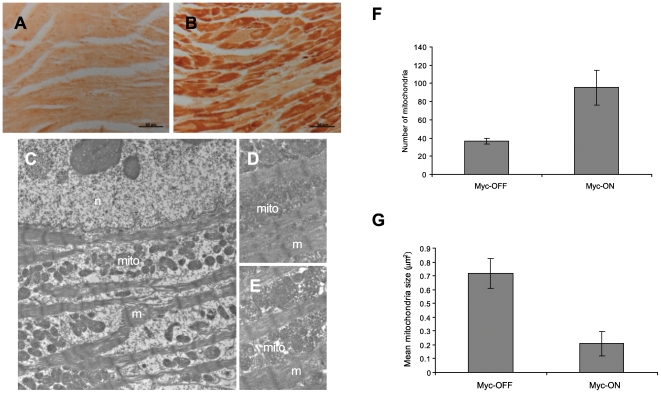
The expression of PGC-1α and mitochondrial alteration in Myc-ON mice. The expression of PGC-1α is examined by PGC-1α specific antibodies. The representative figures demonstrate strong induction of PGC-1α in the cardiomyocytes of Myc-ON mice (B) compared with Myc-OFF mice (A). Low-power electron microscopy reveals displacement of myofibrils in Myc-ON mice (C). The number and density of mitochondria are increased in Myc-ON mice (D) compared with Myc-OFF mice (E). Morphometric analysis demonstrated about 2.5 fold increase of mitochondria number and about 30% decrease of mitochondrial size in the cardiomyocytes of Myc-ON mice (p<0.05) compared with Myc-OFF mice (F). n: nucleus, m: myofibrils, mito: mitochondria. Original magnification: X 400 for A and B, X 30,000 for C, D and E.

To confirm and extend the histological changes, the cellular ultrastructure of ventricular tissue from Myc-ON mice was examined by electron microscopy. Consistent with our histological analyses (Figure 3), Myc-ON mice showed a profound disruption of myofibrillar structure and, interestingly, a dramatic proliferation of mitochondria (Figure 7C,F). Since the disruption of myofibrils broadens the space mitochondria occupy and makes the measurement of total mitochondria in a fixed area difficult, sites containing relatively intact myofibrils were selected for morphometric measurements. Interestingly, increases in mitochondria number and density, but decreases in size, in Myc-ON mice are evident compared to Myc-OFF mice (Figure 7D–G). This result suggests that mitochondrial changes occur earlier than myofibril disruption and other pathological changes. In this regard, it is interesting to note that PGC-1α,a major regulatory molecule of mitochondrial proliferation, is known to play an important role inin hypertrophic cardiomyopathy[19], [20]. To determine the involvement of PGC-1α in Myc-ON mice, we analyzed the expression of PGC-1α and found that the level of PGC-1α was significantly increased in the cardiomyocytes in Myc-ON mice compared with Myc-OFF mice (Figure 7A,B). Taken together, our data suggest that mitochondrial alterations in cardiomyocytes are affected by Myc expression.

### Cardiac mitochondrial functional analysis in Myc mice

**Figure 8:**
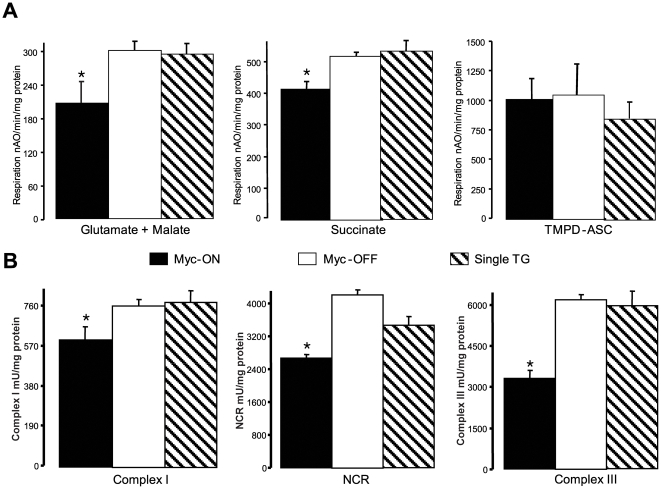
The expression of Myc leads to a decreased rate of dinitrophenol uncoupled respiration when glutamate+malate and succinate+rotenone were used as complex I and complex II substrates compared to the mitochondria from Myc-OFF or single transgenic mice (Single Tg) (Panel A). Myc did not affect the oxidation of TMPD-ascorbate (Panel A). Direct measurement of electron transport chain enzyme activity shows that Myc decreased activities of complex I, NCR (NADH cytochrome *c* oxidoreductase), and complex III (Panel B). Data are expressed as mean±SEM; * p<0.05 vs. Myc-OFF and Single Tg. n = 5 for Myc-ON and Myc-OFF, and n = 4 for Single Tg.

Based on our ultrastructural findings, and since defects in mitochondrial metabolism contribute to the pathogenesis and progression of heart failure, we explored whether functional deficits in mitochondrial metabolism were associated with the observed changes in size and number of mitochondria in Myc-ON mice. Myc-ON expression led to a decrease in protein yield of mitochondria compared to the Myc-OFF or single transgenic mice (Table 2). Moreover, the specific activity of citrate synthase, a mitochondrial marker enzyme, was decreased in the mitochondria from Myc-ON mice compared to the mitochondria from Myc-OFF or single transgenic mice (Myc-ON 3540±160* mU/mg protein, n = 5 , p<0.05 vs. both Myc-OFF 4159±144, n = 5, and Single Tg 4105±156, n = 4). With glutamate plus malate as the donor of reducing equivalents to complex I, the rate of state 3 respiration was lower in Myc-ON compared to the Myc-OFF or single transgenic mice and state 4 respiration was also decreased in Myc-ON mitochondria compared to other groups. In contrast, the respiratory control ratio (RCR, state3/state4) was higher in Myc-ON mouse mitochondria compared to the Myc-OFF or single groups and there was no difference in the ADP/O ratio among groups (Table 2). With succinate plus rotenone as the complex II substrate, respiration was also decreased in Myc-ON mitochondria. The decreased oxidation in the presence of dinitrophenol localizes the defect to the mitochondrial electron transport chain (Table 2, Figure 8).

**Table 2:** The rate of oxidative phosphorylation (nAO/min/mg) in isolated mitochondria with glutamate and malate as complex I substrates.

	Single Tg (n = 4)	Myc-OFF (n = 5)	Myc-ON (n = 5)
Protein yield (mg/g)	17.6±0.3	18.9±1.6	16.5±1.0*
State 3 (0.2 mM ADP)	320±12	323±20	259±21*
State 3 (2 mM ADP)	301±19	321±21	209±31*
Dinitropenol (0.2 mM)	293±19	300±15	206±38*
State 4 (ADP limited)	74±2	69±2	50±9*
RCR (state3/state4)	4.4±0.2	4.7±0.3	6.3±1.7*
ADP/O	2.97±0.06	3.03±0.07	3.12±0.07

Mean±SEM.

* p<0.05 vs. Myc-OFF and Single Tg mice. RCR, respiratory control ratio; DNP, 2 mM ADP was used to measure state 3 respiration under conditions of saturating ADP.

Decreases in glutamate or succinate oxidation can be due to defects at complex III or complex IV (cytochrome oxidase). In this regard, TMPD-ascorbate donates electrons to cytochrome oxidase via cytochrome *c* and, importantly, unchanged TMPD oxidation excludes a defect at complex IV and further implicates upstream complexes (Figure 8). To further localize the defective sites within the electron transport chain, we directly measured the activities of complex I, complex II, and complex III and, consistent with above data, the conditional expression of Myc (Myc-ON) led to decreased activities of complex I and NCR compared to the Myc-OFF or single transgenic mice (Figure 8). NCR measures complex I–III activity with complex I considered to be the rate controlling step and, as such, the decreased rate of NCR supports a complex I defect in the electron transport chain in Myc-ON mice. To further dissect the defect within complex I, we determined the activity of NFR (a proximal part of complex I) [22]. The activity of NFR was unchanged in Myc-ON mice, indicating that the defect is distal to the first part of complex I in Myc-ON mice.

**Figure 9:**
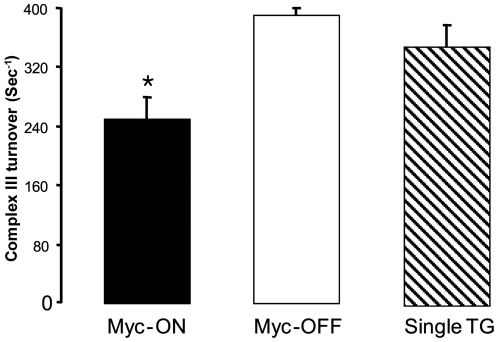
The complex III turnover is decreased in Myc-ON mice (n = 5) compared with the Myc-OFF (n = 5) or single transgenic mice (Single Tg) (n = 4). Complex III turnover (sec^−1^) = complex III activity (nmol/mg protein/sec)/cytochrome b content (nmol/mg protein). Data are expressed as mean±SEM; † p<0.05 vs. Myc-OFF or single transgenic mice (Single Tg).

Complex II activity was not altered by Myc-ON gene expression (data not shown) and the decrease in succinate driven respiration without a change in complex II activity coupled with normal oxidation of TMPD-ascorbate suggests the presence of a defect at complex III [23]. Direct measurements confirmed that the activity of complex III was significantly (p<0.05) decreased in Myc-ON mice (Figure 9). Complex III has three catalytic subunits: cytochromes *b*, *c*_1_ and the iron-sulfur protein and, interestingly, the decrease of cytochrome *b* content without affecting cytochrome *c*_1_ content was observed in Myc-ON mice and the content of cytochrome *c* and *aa*_3_ were also decreased in Myc-ON mice (Table 3). Since each complex III monomer contains a single cytochrome *b* subunit, we calculated the complex III turnover number using the ratio of complex III activity/cytochrome *b* content. The decreased complex III turnover in Myc-ON mice mitochondria suggests that the decrease in respiration through complex III in Myc-ON mice was due to a decreased content of complex III in mitochondria as well as a decreased activity of complex III per each monomer.

**Table 3:** Cytochrome contents (nmol/mg protein) in isolated mouse heart mitochondria.

	*c*	*c*_1_	*b*	*aa*_3_
Single Tg (n = 4)	0.49±0.02	0.18±0.01	0.29±0.01	0.72±0.03
Myc-OFF (n = 5)	0.48±0.01	0.19±0.01	0.27±0.01	0.67±0.03
Myc-ON (n = 5)	0.39±0.02*	0.16±0.01	0.22±0.01*	0.52±0.05*

Mean±SEM.

* p<0.05 or 0.01 vs. Myc-OFF and Single Tg mice.

## Discussion

In this study, to study the pathological role of Myc in cardiomyopathy and heart failure, we developed and characterized a newly established bi-transgenic mouse (MHC-Myc), which expresses Myc specifically in cardiomyocytes in a doxycycline-dependent manner. Since Myc is only expressed temporally during development, not in adult, in cardiomyocytes, the ability to regulate the expression of Myc after development was especially important in this study. In MHC-Myc mice, the expression of Myc is tightly regulated by doxycycline and no significant phenotype is observed until Myc was induced. However, induction of Myc (Myc-ON) for two weeks led to dramatic changes in the heart including an ectopic reactivation and expression of cell cycle markers in cardiomyocytes and mitochondrial perturbations that culminated in severe hypertrophy and, ultimately, death. These results strongly support the notion that increased Myc in adult cardiomyocytes induces cell cycle re-entry that leads to cardiomyopathy and is consistent with a previous report showing that the conditional inactivation of Myc attenuates cardiac hypertrophy induced by pressure overload and other hypertrophic stimuli [24]. However, in contrast to a previous Myc inducible mouse model [25], induction of Myc in MHC-Myc animals ultimately causes heart failure resulting in death in a relatively short duration (2–3 weeks). This difference is likely a consequence of mouse strain and expression level of Myc (Robb MacLellan, personal communication). While our MHC-Myc mouse is derived from FVB strain, the transgenic mouse line used by others [25], [26] is derived from C57Bl/6 and C3H mixed background. Since the effect of genetic background on the development of cardiomyopathy and heart failure is well documented [27], [28], it is likely that genetic factor(s) affect subsequent phenotypes. In addition, our MHC-Myc mouse studies here appear to have significantly higher levels of Myc expression and, consequently, a more robust phenotype than previous models. Supporting this, the number of cardiomyocytes re-entering the cell cycle in our MHC-Myc animals is much greater than reported previously [24], [25]. Therefore, the different phenotypes observed between transgenic mice is likely a consequence of the extent to which cardiomyocytes re-enter the cell cycle and progress into S phase, leading to the development of hypertrophy and consequent heart failure. Supporting this, recent studies have suggested that the adult myocardium is capable of limited cell division in certain pathological conditions [29], [30]. For instance, heart regions adjacent to a myocardial infarction show a significant increase of Ki-67 proliferation index, while no changes were noted distant from the lesion. Additionally, mitotic activity in cardiomyocytes is increased nearly 10-fold increase in end-stage ischemic heart disease and in idiopathic dilated cardiomyopathy [29]. Therefore, our data, in addition to other studies, strongly suggests that the reactivation of cell cycle in adult cardiomyocytes plays an important role in the development of cardiomyopathy and heart failure. This said, further study into the relationship to cell cycle re-entry as an adaptive or maladaptive process is clearly necessary.

Another important finding of our study is that Myc induces the morphological and functional alterations of mitochondria in MHC-Myc mice. Since PGC-1α related pathways can be directly regulated by Myc [18], we suspect that Myc-mediated PGC-1α regulation, which is tightly related with mitochondrial biogenesis and function, is a key pathway for the development of hypertrophic cardiomyopathy and heart failure in MHC-Myc mice [19], [20]. In support of this, cardiac-specific overexpression of PGC-1α, driven by the α-myosin heavy chain promoter, leads to marked mitochondrial proliferation, severe cardiomyopathy and death [19]. Further, of more physiologic relevance, is a model of inducible PGC-1α overexpression in the adult mouse, which leads to a more subtle increase in mitochondrial number and the development of a reversible cardiomyopathy, with both systolic and diastolic functional impairment [20]. The potential mechanism(s) of mitochondrial-mediated cardiac dysfunction by PGC-1α overexpression likely involves defects in mitochondrial oxidative metabolism or the increased production of reactive oxygen species [20]. Consistent with this notion, the increase of PGC-1α in our MHC-Myc mice was concomitant with an increase in mitochondrial number, a decrease in mitochondria size, and a reduction in the activity of mitochondrial oxidative metabolism. While the mechanistic basis for PGC-1α-mediated cardiomyopathy is unclear, it is likely that dysregulated mitochondrial metabolism and/or morphological alteration play an important role in the development of cardiomyopathy and heart failure. Interestingly, a recent study demonstrated an increased level of PGC-1α, but not activity, in failing human hearts suggesting that, despite the increase of PGC-1α, downstream pathways were disturbed by other mechanisms to induce mitochondrial pathologies [31]. Our observations in MHC-MYC mouse are consistent with these data from human patients as we also observed an increase of level of PGC-1α accompanied by functional deficits in mitochondrial energy metabolism.

Notably, it is well established that defects in electron transport chain complexes I [14], complex III [15] and IV [16] lead to dilated cardiomyopathy in human patients. Moreover, the enzyme activity of electron transport chain complexes is decreased in hearts explanted from patients with end-stage heart failure [15], [32]–[36] and, importantly, end-stage heart failure also results in re-expression of Myc as well as decreases in complexes I, III, and IV, and mutations in mtDNA [33], [34]. Therefore, the functional defect in mitochondrial energy metabolism in MHC-Myc mice is likely a key mechanism for the development of cardiac phenotypes such as hypertrophy and heart failure. Regarding this, the smaller size of mitochondria may also explain the defect in mitochondrial function coupled with an increase of mitochondrial number. In fact, our biochemical analyses show that citrate synthase, amount and activity, is decreased in MHC-Myc mice. Since citrate synthase is a mitochondrial matrix marker enzyme, reflecting relative mitochondrial abundance and purity, these results suggest that Myc may increase the number of immature mitochondria. The decrease in cytochrome content and multiple defects in the electron transport chain further supports the notion that mitochondrial development may be incomplete in Myc-ON mice [17]. Collectively, these findings could explain increased mitochondrial number coupled with decreased activities of citrate synthase, complex I, and complex III.

In conclusion, our findings using a novel transgenic mouse with cardiomyocyte-specific expression of Myc (MHC-Myc mice) clearly demonstrate that increased expression of Myc can induce hypertrophic cardiomyopathy and heart failure *in vivo*. Mechanistically, dysregulation of cell cycle and concomitant mitochondrial alteration are likely key pathogenic mechanisms for subsequent hypertrophy-induced heart failure. As such, therapeutics targeted toward Myc, cell cycle re-activation, and/or mitochondrial dysfunction may provide opportunities for the treatment and management of cardiomyopathies.

## Materials and Methods

### Animals

We used the reverse tetracycline transactivator (rtTA) system to generate bi-transgenic mice that inducibly overexpress human c-Myc cDNA under the control of α-myosin heavy chain (MHC) promoter to drive high transgene expression specifically in the cardiomyocyte. All MHC-Myc mice were derived from crossing αMHC-rtTA [21] and tet-o-Myc mice [9], and the genotypes of the F1 progeny confirmed by PCR. All mice in this study were conceived and raised without doxycycline to prevent any potential developmental consequences from the expression of Myc. To induce Myc expression in cardiomyocytes, doxycycline-containing food (200 mg/kg; Bio-Serve, Frenchtown, NJ) was provided *ad libitum* to animals and sequential echocardiograms were performed. Thereafter, animals were sacrificed for further pathophysiological analysis. Importantly, in preliminary experiments, we found that the doxycycline diet had no effect on single transgenic littermates, which were used as a single transgenic mouse control, on the development of cardiac hypertrophy nor other biochemical analyses performed in this study. All study protocols were approved by Case Western Reserve University School of Medicine Institutional Animal Care and Use Committee.

### Immunocytochemistry

Formalin fixed hearts were processed and embedded in paraffin. Six µm thick serial sections were cut, mounted onto slides and rehydrated according to standard protocols. Immunocytochemistry was performed by the ABC method according to the manufacturer's protocol (Vector Lab, Burlingame, CA). Anti-PCNA mouse monoclonal antibody (Santa Cruz Biotech, Santa Cruz, CA), anti-Myc rabbit monoclonal antibody (Epitomics, Burlingame, CA), anti-Ki67 mouse monoclonal antibody (Dako, Carpinteria, CA), anti-PGC-1α rabbit polyclonal antibody (Santa Cruz Biotech, Santa Cruz, CA) and anti-Cyclin D1 rabbit antibody (Thermo Scientific, Waltham, MA) were used. Antibody reactivity was localized using 3-3′-diaminobenzidine as a chromogen (Dako, Carpinteria, CA). To exclude the possibility of non-specific reaction, all the immunocytochemistry experiments contained at least one sample without a primary antibody. Haemotoxylin and eosin staining and Trichrome staining were also performed for routine histochemical and morphological analyses. Quantification of Ki67- and BrdU-positive cardiomyocytes was performed on slides stained and counterstained with haematoxylin. Four areas of kleft venticles were randomly selected on an Axiophot microscope (Zeiss, Thornwood, NY) and at least 100 positive cardiomyocytes were counted per area. Percent positive was calculated by dividing the percent positive nuclei by total number of cardiomyocytes counted per experimental group (n = 3).

### RT-PCR

mRNA was isolated from heart tissues using an RNeasy kit (Qiagen, Valencia, CA). RT-PCR was carried out using the RETROscript RT-PCR kit (Ambion, Austin, TX) according to the manufacturer's instructions. Briefly, purified total RNA (100 ng) was reverse transcribed with cloned MMLV reverse transcriptase (50 U) by incubating at 44°C for 60 min, followed by heating at 92°C for 10 min. The resulting single-stranded cDNA was then amplified using a pair of primers for each target gene (25 pmol) and Taq DNA polymerase (2.5 U; Roche, Indianapolis, IN) for the indicated number of cycles of amplification (30 sec at 94°C for denaturing, 30 sec at 61°C for primer annealing, and 30 sec at 72°C for primer extension). The RT-PCR products were then subjected to electrophoresis in a 1.5% agarose gel. The nucleotide sequence used for each primer was as follows: for c-Myc: 5′-primer, 5′-TCTGGATCACCTTCTGCTGG-3′; 3′-primer, 5′-CCTCTTGACATTTCTCCTCGG-3′, for atrial natriuretic peptide: 5′-primer, 5′-CATCACCCTGGGCTTCTTCCT-3′; 3′-primer, 5′-TGGGCTCCAATCCTGTCAATC-3′, for brain natriuretic peptide: 5′-GCG GCA TGG ATC TCC TGA AGG-3′; 3′-primer, 5′-CCC AGG CAG AGT CAG AAA CTG-3′, and for GAPDH: 5′-primer, 5′-ATGTTCCAGTATGACTCCACTCAGG-3′; 3′-primer, 5′-GAAGACACCAGTAGACTCCACGACA-3′.

### TUNEL analysis

Detection of 3′-OH termini of DNA strand breaks was performed on paraffin sections using the In Situ Cell Death Detection Kit (Roche, Indianapolis, IN) following the recommendations of the manufacturer. Briefly, the tissue sections were treated with proteinase K (20 µg/ml in 10 mM Tris-HCl, pH 7.4) for 30 min at 37°C after rehydration. After rinsing slides with phosphate buffered saline, TUNEL reaction mixture containing terminal deoxynucleotidyl transferase and fluorescence labeled nucleotide was applied for 1 hr at 37°C. Positive signals were observed directly using fluorescence microscopy. Positive and negative controls were run in parallel on adjacent sections to confirm the specificity of the assay.

### BrdU incorporation analysis

DNA synthesis was directly examined by analysis of 5-bromo-2′-deoxyuridine (BrdU) incorporation. For this experiment, 50 mg/kg of the thymidine analog BrdU (Sigma, St. Louis, MO) was intraperitoneally injected into each mouse. One day after injection, hearts were removed and embedded in paraffin, sectioned, and mounted on slides. The sections were treated with 2N hydrochloric acid for 2 hr and then washed two times with 1x Tris-buffered saline (3 min each) to neutralize the acid. Incorporated BrdU was detected with an anti-BrdU rat monoclonal antibody (Abcam, Cambridge, MA) diluted 1∶200 by the ABC method as described above.

### Electron microscopy

Electron microscopy on tissue sections was performed as described previously [37], [38]. Briefly, tissue was immediately fixed in 1.5% glutaraldehyde in cacodylate buffer, pH 7.4 for 1 hour at room temperature and postfixed in 1% osmium tetroxide for 1 hour. After dehydration in graded ethanol and propylene oxide, the tissue was embedded in epoxy resin, then sectioned at silver interference color. Thin sections were stained with uranyl acetate and lead citrate, and examined in a JEOL 1200EX electron microscope (Tokyo, Japan).

### Echocardiographic analysis

Mice were weighed and anesthetized with isoflurane (induced at 3% and maintained at ∼1% by nosecone). After shaving the chest, the extremities were secured to a warming pad (Braintree Scientific, Braintree, MA) with paper tape and needle electrodes were connected to a preamplifier to simultaneously record a single lead electrocardiogram. Pre-warmed, centrifuged (to eliminate air bubbles) ultrasound transmission gel was applied to the chest and 2D-directed M-mode and Doppler echocardiographic studies were performed using a 15 MHz (15L8) linear array transducer (Sequoia, Siemens, Mountain View, CA). Mice were imaged in the shallow left lateral decubitus position and short and orthogonal long axis and apical views were obtained. The M-mode dimensions included the end-diastolic (LV EDD) and end-systolic (LV ESD) diameters [taken from the short axis at the level of the largest left ventricle (LV) diameter], end-diastolic posterior (EDPWTh) and end-diastolic septal (EDSWTh) wall thicknesses, the LV outflow tract diameter (LVOT) and left atrial and, aortic root dimensions. Doppler variables included the peak and velocity time integral (vti) of aortic flow, and aortic ejection time. Simultaneous transmitral and LV outflow velocities were obtained for calculation of the relatively heart rate-independent myocardial performance index (MPI), which is a measure of combined systolic and diastolic function [39]. The MPI is the sum of the isovolumic contraction and relaxation times divided by the aortic ejection time. Other derived echo indices included the LV fractional shortening (FS = (EDD-ESD)/EDD), LV mass [LVM = 1.06*(EDD+EDPWTh*EDSWTh)^3^-EDD^3^)], relative wall thickness [RWT = (EDWTh+EDSWTh)/EDD)], and cardiac output [CO = (LVOT/2)^2^*aortic vti*heart rate]. LV mass was normalized by the body weight. Studies were performed by a sonographer who was blinded to genotype.

### Mitochondria isolation

A single population of cardiac mitochondria, consisting of a combination of subsarcolemmal and interfibrillar mitochondria, was isolated using protease treatment. Cardiac tissue from a pool of 3 to 5 mouse hearts was finely minced and placed in buffer A [containing (in mM) 100 KCl, 50 MOPS, 1 EGTA [ethylene glycol-bis (β-aminoethyl ether)-N,N,N′,N′-tetraacetic acid], 5 MgSO_4_•7 H_2_O, and 1 ATP; pH 7.4] at 4°C. The minced tissue was homogenized and incubated for 30 sec at 4°C in the presence of protease (5 mg/g wet tissue). Buffer B (Buffer A+0.2% bovine serum albumin) was added to neutralize the protease reaction. The mixed homogenate was quickly centrifuged at 8,000 g for 10 min, and the supernatant was discarded. The pellets were re-suspended in buffer B, centrifuged at 500 g for 10 min, and the supernatant saved for mitochondria isolation. The pellets were resuspended one more time, washed in Buffer B, re-centrifuged, and the remaining pellet discarded. The combined supernatants were centrifuged at 3,000 g to sediment mitochondria, which were sequentially washed with buffer B followed by KME (100 mM KCl, 50 mM MOPS, and 0.5 mM EGTA). Mitochondria were suspended in KME and the protein content was determined by the biuret method using bovine serum album as a standard [23].

### Mitochondrial oxidative phosphorylation and oxidase activities

Oxygen consumption in mitochondria was measured using a Clark-type oxygen electrode at 30°C [23]. Mitochondria were incubated in a buffer [containing (in mM) 80 KCl, 50 MOPS, 1 EGTA, 5 KH_2_PO_4_, and 1 mg/ml BSA, at pH 7.4]. Glutamate+malate (complex I substrate), succinate (complex II substrate), and TMPD (N,N,N′,N′ tetramethyl p-phenylenediamine)-ascorbate (complex IV substrate) were used as donors to specific sites in the electron transport chain. Rotenone (5 uM) was used with succinate and TMPD-ascorbate to prevent reverse electron flow. State 3 (ADP-stimulated), state 4 (ADP-limited) respiration, respiratory control ratio, and the ADP/O ratio were measured [23]. Dinitrophenol (0.2 mM) was used to determine the maximal rate of uncoupled respiration independent of the phosphorylation apparatus including complex V.

### Electron transport chain and citrate synthase enzyme activities

The following enzyme activities were measured at 37°C in detergent-solubilized mitochondria using previously described methods [40]–[42]: NADH-cytochrome *c* reductase (NCR), rotenone-sensitive; NADH-decylubiquinone reductase, rotenone sensitive (complex I); NADH ferricyanide reductase (NFR); Succinate-decylubiquinone reductase (complex II); Decylubiquinol cytochrome c oxidoreductase (complex III); and citrate synthase.

### Statistics

Statistical comparisons were performed by parametric analysis using a one-way analysis of variance (ANOVA) and/or Students' t-test, as appropriate, to determine significant differences across experimental groups. The null hypothesis was rejected at p<0.05.
